# Methylobacter arcticus sp. nov. isolated from a coal mine biofilm in the high Arctic Svalbard

**DOI:** 10.1099/ijsem.0.006984

**Published:** 2025-11-28

**Authors:** Antti J. Rissanen, Anne Grethe Hestnes, Ramita Khanongnuch, Alena Didriksen, Tim Urich, Alexander T. Tveit, Mette M. Svenning

**Affiliations:** 1 Faculty of Engineering and Natural Sciences, Tampere University, Tampere, Finland; 2 Natural Resources Institute Finland, Helsinki, Finland; 3 Department of Arctic and Marine Biology, UiT, The Arctic University of Norway, Tromsø, Norway; 4 Department of Geography, Institute of Ecology and Earth Sciences, University of Tartu, Tartu, Estonia; 5 Institute of Microbiology, Center for Functional Genomics of Microbes, University of Greifswald, Greifswald, Germany

**Keywords:** Arctic, coal mine, methane oxidation, methanotroph, *Methylobacter*, psychrotrophic

## Abstract

An aerobic methanotroph was isolated from a biofilm of coal mine Gruve 7 (Svalbard) and designated strain G7^T^. Cells of strain G7^T^ were Gram-stain-negative, pink-pigmented and motile rods. Strain G7^T^ could grow at pH 6.8 and at temperatures ranging from 4 to 21 °C. The genome size was 4.00 Mb with a (digital) DNA G+C content of 47.7 mol%. Strain G7^T^ represents a member of the family *Methylomonadaceae* of the class *Gammaproteobacteria*. It displayed 94.6–99.7% 16S rRNA gene sequence similarity to the type strains of the genus *Methylobacter*. Whole-genome comparisons based on average nucleotide identity (ANI) and digital DNA–DNA hybridization (dDDH) confirmed that strain G7^T^ represents a novel species. It showed 16S rRNA gene identity of 99.7%, 91.8% ANI and 46% dDDH to the closest type strain, *Methylobacter svalbardensis* LS7-T4A^T^, with ANI and dDDH being much lower than the typically used 95 and 70% cutoffs, respectively, to delineate different species. For methane activation, strain G7^T^ carries genes encoding particulate methane monooxygenase (pmoCAB). Also, genes of the methane utilization pathways, i.e. oxidation of methane to carbon dioxide and assimilation of methane-carbon to biomass, were encoded in the genome. Strikingly, compared to all other *Methylobacter* spp. strains, strain G7^T^ did not have nitrogenase genes for nitrogen fixation. Strain G7^T^ also possessed genes for ectoine production, which was not observed in the genomes of its closest relatives. Based on phenotypic, genetic and phylogenetic data, strain G7^T^ represents a novel species within the genus *Methylobacter* for which the name *Methylobacter arcticus* sp. nov. is proposed, with strain G7^T^ (DSM: 117899; LMG: 33632) as the type strain.

## Introduction

*Methylobacte*r is a genus of methane-oxidizing bacteria (methanotrophs). It belongs to the family *Methylomonadaceae* of the class *Gammaproteobacteria* in the phylum *Pseudomonadota*. *Methylobacter* species are ubiquitous in nature, acting as biofilters for methane in diverse natural aquatic and terrestrial environments, as well as in anthropogenic ecosystems [1,2]. They have been enriched and isolated from natural ecosystems such as tundra soil, wetland soil, freshwater and estuary sediments, as well as from lake water and anthropogenic methane-emitting ecosystems, such as landfills and rice fields (Table 1). The current type species include *Methylobacter whittenburyi* 1521^T^, *Methylobacter marinus* A45^T^, *Methylobacter luteus* NCIMB 11914^T^, *Methylobacter psychrophilus* Z-0021^T^, *Methylobacter tundripaludum* SV96^T^ and *Methylobacter svalbardensis* LS7-T4A^T^ which have been isolated from wetlands, sediments, mud and soils (Table 1). The type species represent different cell morphologies, i.e. cocci and rods, and temperature preferences, i.e. mesophilic, psychrotolerant and psychrophilic species.

**Table 1.** Similarity of the 16S rRNA gene (% similarity), *pmoA* gene [% similarity at nucleotide level (amino acid level)] and genome, i.e. average nucleotide and amino acid identity (ANI and AAI in %) and digital DNA–DNA hybridization (dDDH in %), of strain G7^T^ with those of reference strains (isolation/enrichment source and database accession numbers also shown)

Strain G7^T^ compared to	Source	16S rRNA gene acc.	Genome acc.	16S rRNA gene	*pmoA* gene	ANI	AAI	dDDH
*M. psychrophilus* FCN1	Tailings pond water	OK135604.1	No data	99.6	No data	No data	No data	No data
*Methylobacter* sp. NLS-2M	Landfill soil	OM535268.1	No data	99.6	No data	No data	No data	No data
*Ca*. Methylobacter titanis K-2018	Antarctic lake sediment	From genome	GCA_029945785.1	99.6	97.45 (98.79)	95.7	94.3	63.6
*Ca*. Methylobacter titanis D1-2020	Antarctic lake sediment	From genome	GCA_029946125.1	99.6	97.45 (98.79)	95.7	94.7	63.9
*M. svalbardensis* LS7-T4A^T^	Arctic Lagoon Pingo sediments	OQ832782.1	GCA_037671525.1	99.7	99.19 (100)	91.8	89.0	46
*M. tundripaludum* SV96^T^	Arctic wetland soil	NR_042107.1	GCA_000190755.3	98.7	94.35 (97.57)	85.3	82.4	30.5
*M. tundripaludum* OWC-DMM	Wetland	From genome	GCA_002934385.1	98.7	94.35 (97.57)	85.2	82.6	30.2
*M. tundripaludum* 21/22	Lake sediment	From genome	GCA_000685925.1	98.5	94.62 (97.98)	85.2	82.5	30.5
*M. tundripaludum* 31/32	Lake sediment	From genome	GCA_000733835.1	98.5	93.55 (97.98)	85.2	82	30.4
*Ca*. Methylobacter oryzae KRF1	Tropical rice field	MK511847.1	GCA_003994235.2	97.4	90.46 (93.93)	80.8	78.3	24.1
*Methylobacter* sp. S3L5C	Boreal lake water	OM479427.1	GCA_022788635.1	98.7	90.95 (93.9)	79.0	72.0	22.4
*M. psychrophilus* Z-0021^T^	Arctic tundra soil	NR_025016.1	GCA_025583945.1	98.7	89.25 (91.87)	78.4	71.9	22
*M. luteus* NCIMB 11914^T^	Fresh water mud	NR_041814.1	GCA_000427625.1*	96.2	87.25 (91.9)	78.0	70.8	21.2
*M. marinus* A45^T^	Sewage outfall sediment	NR_025132.1	GCA_000383855.1	96.0	87.13 (92.71)	77.5	70.1	20.5
*M. whittenburyi* 1521^T^	Estuary sediment	NR_029242.1	GCA_000746145.1†	94.6	87.13 (92.71)	77.4	70.2	20.5
*Ca*. Methylobacter coli	Faeces (Indian antelope)	From genome	GCA_015476545.1	95.9	85.43 (91.09)	77.3	70.3	20.6
*Ca*. Methylobacter favarea B2	Volcanic soil	No data	GCA_902806695.1	no data	82.58 (87.04)	77.2	71.2	20.7

*Genome represents strain IMV-B-3098, as the genome of the type strain was not found.

†Genome represents strain BBA5.1, as the genome of the type strain was not found.

Coal mines and coal mining represent a significant anthropogenic methane source, accounting for 33% of total fossil fuel–related emissions of methane (for the 2008–2017 decade) [3]. Culture-independent studies have revealed methanotrophic activity and several methanotrophic bacterial genera (incl. *Methylobacter*) in rocks of coal mines [4,7], yet cultured representatives of coal mine methanotrophic bacteria are lacking. In our preliminary experiments, we noticed active CH_4_ consumption by coal samples from the coal mine Gruve 7 (Fig. S1, available in the online Supplementary Material). As a result, we enriched and isolated a methanotrophic bacterium, strain G7^T^, from the biofilm on the coal. In our previous study focusing on thermal acclimation of *Methylobacter* methanotrophs, strain G7^T^ was studied for its temperature preferences and cellular fatty acids [8]. Here, we extend the characterization of G7^T^ to include other phenotypic properties, as well as phylogenetic and genomic data. Based on the results of phenotypic, genetic and phylogenetic analyses, we propose that strain G7^T^ represents a novel species within the genus *Methylobacter*.

## Methods

### Enrichment, isolation and cultivation

A greyish biofilm was sampled from a humid area on the wall, deep within the coal mine Gruve 7, on 3 August 2009. Gruve 7 is located at 78° 09′ 24″ N 16° 01′ 23″ E in Adventdalen nearby Longyearbyen, Svalbard. The air within the mine normally has a CH_4_ concentration of 0.1% and an ambient temperature of 10–11 °C (personal communication staff coal mine Gruve 7). The mine is set to close for the extraction of coal in summer 2025.

Pieces of coal containing biofilm were transported in a cooling bag to the laboratory. Samples of the biofilm were transferred to 120 ml serum bottles and re-suspended in 20 ml of 10× diluted nitrate mineral salts (NMS) medium (DSMZ medium 921, pH 6.8) in 120 ml serum bottles sealed with rubber septa and aluminium crimp caps. CH_4_ was added to the air in the bottle headspace to a final concentration of 20% (v/v) and incubated in the dark at 10 °C. When visible growth was observed, subsamples were transferred to new 120 ml serum bottles and plated on 1/10 NMS agar plates that were also incubated in containers with 20% CH_4_ (v/v) in air in the dark. Colonies from agar plates were used to inoculate new serum bottles. Transfer between liquid and solid culture was repeated several times. A small aliquot was also plated on nutrient-rich medium TYGA (5 g Tryptone/Peptone, 2.5 g Yeast extract, 1 g Glucose and 20 g Agar per 1 l Tap water) and incubated without CH_4_ to check for the presence of heterotrophic contaminants. The purity of the culture was confirmed when one cell type was observed under a light microscope, and growth was absent on TYGA. The pure culture was attained in 2012. The strain G7^T^ was preserved as an active culture and cryopreserved in 7% DMSO. Storage via lyophilization has not been tested.

### 16S rRNA gene and genome analysis

The standard CTAB extraction protocol of the DOE Joint Genome Institute [9] was used for the genomic DNA (gDNA) isolation from cells of strain G7^T^ that had been grown in liquid culture. The 16S rRNA genes were amplified from gDNA using primers 27F (AGAGTTTGATCMTGGCTCAG) and 1492R (TACGGYTACCTTGTTACGACTT) and sequenced using primer pairs 785F (GGATTAGATACCCTGGTA) and 907R (CCGTCAATTCMTTTRAGTTT) by Sanger Sequencing at UiT, The Arctic University of Norway (Tromsö, Norway). A 16S rRNA gene-based alignment followed by a phylogenetic tree was constructed in Mega 11 using the maximum-likelihood algorithm (generalized time-reversible model) with 100 bootstraps [10].

The genome sequencing and assembly service was provided by the Norwegian Sequencing Centre (https://www.sequencing.uio.no/). DNA of strain G7^T^ was fragmented to 10–15 kb fragments using g-tubes (Covaris). The library was prepared using the Pacific Biosciences protocol for HiFi library prep using SMRTbell^®^ ExpressTemplate Prep Kit 3.0. Sequencing was done, following the Microbial Assembly – mode, on the part of 25M SMRT cell on Revio instrument using Revio Polymerase kit and sequencing plate. Circular consensus sequencing analysis for generation of HiFi reads was performed on the instrument (ICS SW v. 13.0.0.212033). Altogether, there were 8,639 HiFi reads with an average length of 8,003 bp. *De novo* assembly was done using IPA HiFi genome assembler (https://github.com/PacificBiosciences/pbipa) including genome polishing using Racon [11].

Basic statistics of the strain G7^T^ genome and reference genomes were calculated using Prokka (v. 1.14.6) [12]. The genomes were functionally annotated for the presence or absence of genes, functional traits and KEGG modules, using METABOLIC (v. 4.0) [13]. The general differences in the functional potential between *Methylobacter* spp. species were assessed via UPGMA cluster analysis of the presence–absence data of functional traits and KEGG modules encoded in the genomes. Secondary metabolite biosynthesis gene clusters were also identified and annotated using antiSMASH (v. 8.0.2) [14]. The genome of strain G7^T^ and its closest cultured representative (*Methylobacter svalbardensis* LS7-T4A^T^) were also specifically annotated according to the KEGG system using KofamKOALA [15]. The *pmoA* genes encoding the beta subunit of particulate methane monooxygenase (pMMO) were extracted from genomes of G7^T^ and reference genomes using Prokka (v. 1.14.6) [12]. They were then translated to amino acid sequences, which were aligned and subjected to phylogenetic tree analysis using the neighbour-joining method (Jones-Taylor-Thornton model) with 500 bootstrap replicates in Mega 11 [10]. The genome-wide phylogenetic tree was built from protein alignments produced in PhyloPhlAn (v. 3.0.67; PhyloPhlAn database including 400 universal marker genes; ‘-diversity low’ - argument) [16,17] using the maximum-likelihood algorithm (PROTCATLG − model) with 100 bootstrap replicates in RAxML (v. 8.2.12) [18]. Average nucleotide identities (ANI) with reference genomes were calculated using the ANI calculator (http://enve-omics.ce.gatech.edu/ani/, accessed on May 2024) [19]. Average amino acid identities were calculated using the AAI calculator (http://enve-omics.ce.gatech.edu/aai/, accessed in May 2024) [20]. Digital DNA–DNA hybridization (dDDH) comparisons with reference genomes were done using the Type Strain Genome Server (TYGS) online service (https://tygs.dsmz.de/, accessed in May 2024) [21].

### Morphological, physiological and chemotaxonomic characterization

Cell morphology and size of strain G7^T^ were examined via transmission electron microscopy (TEM) and scanning electron microscopy (SEM). A gammaproteobacterial methanotroph membrane structure was confirmed by TEM. Analyses of phospholipid fatty acids (PLFAs) of strain G7^T^ were carried out by the Identification Service, Leibniz-Institut DSMZ – Deutsche Sammlung von Mikroorganismen und Zellkulturen GmbH, Braunschweig, Germany, and the results were reported earlier [8]. The growth response and methane uptake response of strain G7^T^ towards different temperatures (i.e. 4 °C, 8 °C, 15 °C, 21 °C and 27 °C) were also reported previously [8]. Here, we additionally report results on growth and methane uptake at 23 °C. Growth using methanol was determined by incubating G7^T^ at different methanol concentrations (0, 0.01%, 0.05%, 0.1%, 0.5% and 1%) without CH_4_ for 36 days in conditions described above for growth with CH_4_. Similarly, G7^T^ was incubated without nitrate in the medium. In addition, the growth of G7^T^ was compared between NMS and ammonium mineral salts media. Salt tolerance was tested in 1/10 NMS medium (pH 6.8) with the addition of 0, 0.5, 1, 2 and 3% w/v NaCl, and pH optimum using a range from pH 4.5 to 10. No tests of growth on multi-C substrates were performed, as previous studies of *Methylobacter* did not indicate the utilization of such substrates [22,24].

## Results and discussion

### 16S rRNA and *pmoA* gene analysis

**Fig. 1.**
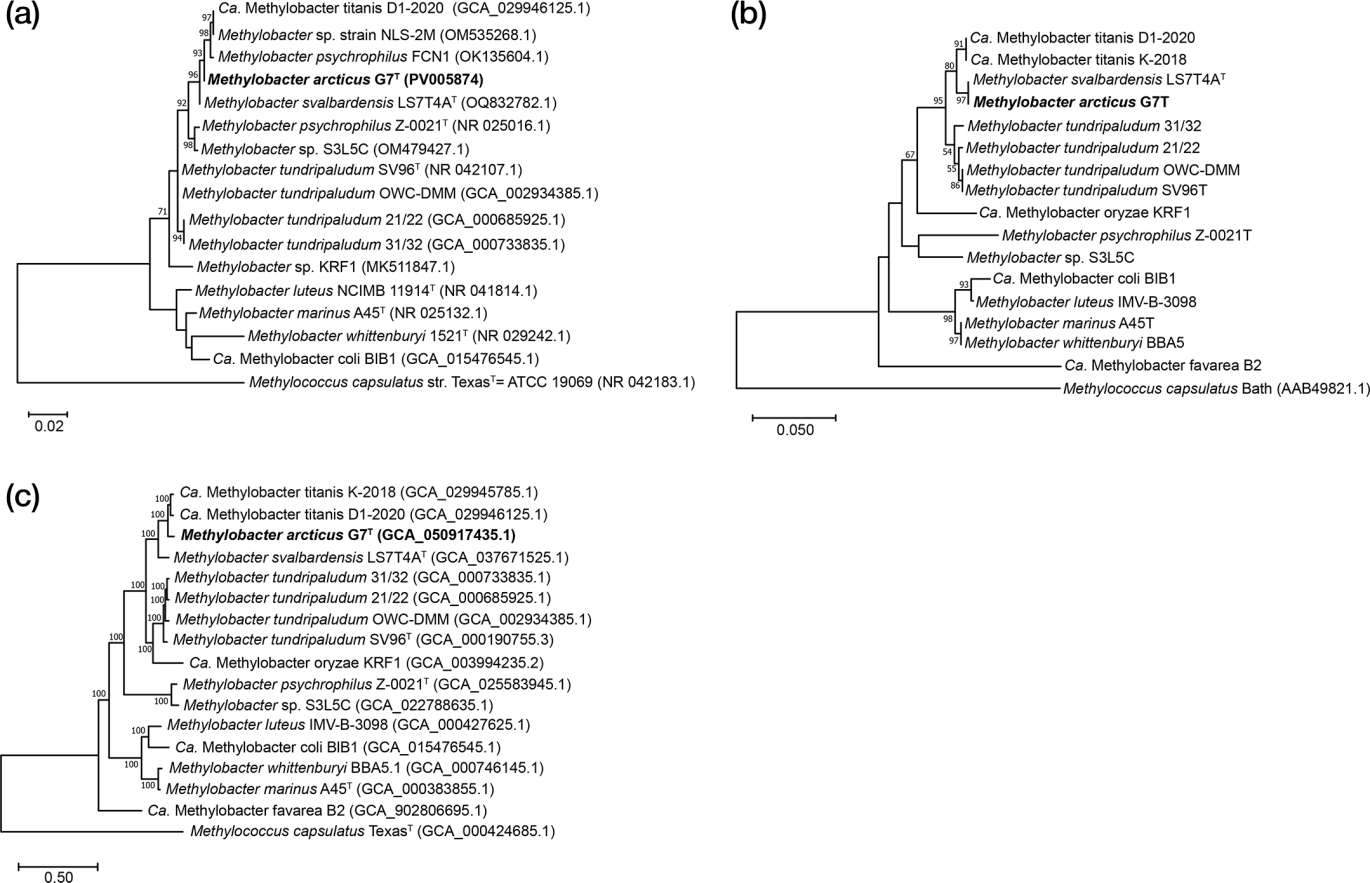
(**a**) Phylogenetic tree based on 16S rRNA genes and (**b**) deduced amino acid sequences of *pmoA* genes, as well as (**c**) genome-wide phylogenomic tree (PhyloPhlAn). 16S rRNA gene and phylogenomic trees were constructed using the maximum-likelihood algorithm, while the *pmoA* gene tree was constructed using the neighbour-joining method, with the GTR model for the 16S rRNA gene tree (in a), the JTT model for the *pmoA* gene tree (in b) and the PROTCATLG model for the genome-wide tree (in c). Numbers at the nodes indicate the percentage of occurrence in 100 bootstrapped trees. The scale bars indicate the number of substitutions per nucleotide (in a) and amino acid position (in b and c). Strain G7^T^ is highlighted in bold. The *pmoA* genes (in b) do not have accession numbers because they were extracted from genomes (see the accession numbers for genomes in c).

Based on a comparison of 16S rRNA genes, strain G7^T^ was most similar, with 99.7% similarity with the recently described psychrophilic strain *M. svalbardensis* LS7-T4A^T^, which was isolated from a terrestrial Arctic alkaline methane seep in Lagoon Pingo, in Central Spitsbergen (Table 1, Fig. 1a) [22]. Furthermore, strain G7^T^’s 16S rRNA gene had 99.6% similarity, with metagenome-assembled genome (MAG) of *Ca*. Methylobacter titanis, from a psychrophilic methanotrophic enrichment culture originating from freshwater lake sediments of Antarctica [25]. In addition, high similarity was identified with 16S rRNA genes of *M. psychrophilus* FCN1 and *Methylobacter* sp. NLS-2M, isolated from tailings pond water and temperate landfill cover soil, respectively, and for which genome sequences are not available (Table 1, Fig. 1a). The next closest strains, with 16S rRNA gene identity of 98.5–98.7%, were the psychrophilic *Methylobacter* sp. S3L5C and *M. psychrophilus* Z-0021^T^ and the various strains of *M. tundripaludum* (Table 1, Fig. 1a) [23,24, 26,28]. In general, the 16S rRNA gene similarity of strain G7^T^ with other *Methylobacter* spp. strains varied between 94.6 and 99.7% (Table 1, Fig. 1a).

As with the 16S rRNA gene comparison, the *pmoA* gene of strain G7^T^ was most similar, with 99.2 and 100% nucleotide and amino acid sequence identity, respectively, with *M. svalbardensis* LS7-T4A^T^. It was less similar to the two MAGs representing *Ca*. M. titanis, with averagely 97.5 and 98.8% nucleotide and amino acid identity, and to *M. tundripaludum* strains, with 93.6–94.6% and 97.6–98.0% nucleotide and amino acid identity, respectively (Table 1, Fig. 1b). In contrast with 16S rRNA gene similarities, the similarity of the *pmoA* gene of G7^T^ was distinct from those of *Methylobacter* sp. S3L5C, with 91.0 and 93.9% nucleotide and amino acid sequence identity, respectively, and *M. psychrophilus* Z-0021^T^, with respective 89.3 and 91.9% identity (Table 1, Fig. 1b). In general, the *pmoA* gene identity of strain G7^T^ with other *Methylobacter* spp. strains varied between 82.6–99.2% and 87.0–100% at nucleotide and amino acid sequence levels, respectively (Table 1, Fig. 1b).

### Genome analysis

The full statistics of *de novo* assemblies and genome characteristics of strain G7^T^, as well as other *Methylobacter* spp. isolates and MAGs, are reported in Table 2. The draft genome of strain G7^T^ consisted of 1 contig, with 4,003,569 bp in total length, G+C content of 47.7 mol%, 3,804 CDSs, 9 rRNA and 45 tRNA genes. The genome quality was very high as shown with high genomic completeness (99.4%) and low contamination (3.1%), assessed using CheckM (v1.2.2, Methylococcales.ms marker set) [29], the criteria for very high quality being >95% and <5%, respectively [30].

**Table 2.** Genome characteristics (size, number of CDSs, tRNAs and rRNAs and G+C content), genes encoding methane and methanol oxidation, as well as N_2_ fixation, and estimated genomic completeness and contamination of genomes of *Methylobacter* sp.

Strain	Size (bp)	Contigs	CDS	tRNA	5S rRNA	16S rRNA	23S rRNA	GC%	CH_4_ oxid.	CH_3_OH oxid.	N_2_ fix.	Compl. (%)	Contam. (%)
*M. arcticus* G7^T^	4003569	1	3804	45	3	3	3	47.7	pmoCAB^1^	mxaFIJGACKLD^2^, xoxF^3^	–	99.43	3.11
*Ca*. M. titanis K-2018 (GCA_029945785.1)	3468869	155	3149	39	1	1	1	47.9	pmoCAB	mxaFIJGACKLD, xoxF	–	98.9	1.82
*Ca*. M. titanis D1-2020 (GCA_029946125.1)	3510200	146	3209	41	1	1	1	47.7	pmoCAB	mxaFIJGACKLD, xoxF	–	99.24	1.58
*M. svalbardensis* LS7-T4A^T^ (GCA_037671525.1)	4316257	220	4072	42	1	1	1	47.9	pmoCAB	mxaFIJGACKLD, xoxF	nifDKH^4^	99.72	0.63
*M. tundripaludum* 21/22 (GCA_000685925.1)	4665210	1	4176	45	3	4	3	49.5	pmoCAB (pxmABC)^5^	mxaFIJGACKLD, xoxF	nifDKH	99.8	1.32
*M. tundripaludum* SV96^T^ (GCA_000190755.3)	4848329	3	4412	45	3	2	3	49.5	pmoCAB (pxmABC)	mxaFIJGACKLD, xoxF	nifDKH	99.8	0
*M. tundripaludum* OWC-DMM (GCA_002934385.1)	4607785	27	4087	41	1	1	1	49.5	pmoCAB (pxmABC)	mxaFIJGACKLD, xoxF	nifDKH	99.8	0
*M. tundripaludum* 31/32 (GCA_000733835.1)	5047546	2	4605	45	3	3	3	49.2	pmoCAB (pxmABC)	mxaFIJGACKLD, xoxF	nifDKH	99.8	1.7
*Ca*. M. oryzae KRF1 (GCA_003994235.2)	5071213	121	4431	40	1	1	1	49.3	pmoCAB (pxmABC)	mxaFIJGACKLD, xoxF	nifDKH	98.88	0.49
*M*. sp. S3L5C (GCA_022788635.1)	4815745	1	4342	48	5	5	5	43.3	pmoCAB, mmoXYZBCD^6^	mxaFIJGACKLD, xoxF	nifDKH	99.38	2.45
*M. psychrophilus* Z-0021^T^ (GCA_025583945.1)	4691082	156	4076	41	2	1	1	43.1	pmoCAB, mmoXYZBCD	mxaFIJGACKLD, xoxF	nifDKH	99.38	1.24
*M. luteus* IMV-B-3098 (GCA_000427625.1)	5029135	4	4571	49	3	3	3	51.1	pmoCAB (pxmABC)	mxaFIJGACKLD, xoxF	nifDKH	99.4	0.14
*M. marinus* A45^T^ (GCA_000383855.1)	4988792	2	4486	50	3	3	3	52.7	pmoCAB (pxmABC)	mxaFIJGACKLD, xoxF	nifDKH	98.5	1.58
*M. whittenburyi* BBA5.1 (GCA_000746145.1)	5074273	88	4567	43	3	1	1	52.2	pmoCAB (pxmABC)	mxaFIJGACKLD, xoxF	nifDKH	98.81	1.28
*Ca*. M. coli BlB1 (GCA_015476545.1)	4865836	227	4397	40	1	1	1	51.3	pmoCAB	mxaFIJGACKLD, xoxF	nifDKH	99.55	0.78
*Ca*. M. favarea B2 (GCA_902806695.1)	4073239	134	3783	36	nd	nd	nd	47.2	pmoCAB	mxaFJD, xoxF	nifDKH	99.21	0.34

(1) pmoCAB=particulate methane monooxygenase, (2) mxaFIJGACKLD=calcium-dependent methanol dehydrogenase, (3) xoxF=lanthanide-dependent methanol dehydrogenase, (4) nitrogenase, (5) pxmABC=a sequence divergent particulate monooxygenase with currently unknown function and (6) mmoXYZBCD=soluble methane monooxygenase.

In contrast to 16S rRNA gene analyses, where strain G7^T^ had the highest similarity to *M. svalbardensis* LS7-T4A^T^, the genomic comparisons showed that strain G7^T^ was most similar with the MAGs representing *Ca*. M. titanis, sharing 95.7% ANI, 94.3–94.7% average amino acid identity (AAI) and 63.6–63.8% dDDH (Table 1, Fig. 1c). Of isolated *Methylobacter* spp., strain G7^T^ was most similar to *M. svalbardensis* LS7-T4A^T^, sharing 91.8% ANI, 89.0% AAI and 46% dDDH (Table 1, Fig. 1c). The G+C content of the genomes of strain G7^T^, *M. svalbardensis* LS7-T4A^T^ and *Ca*. M. titanis was also similar, at 47.7–47.9%, differing from other *Methylobacter* spp. (Table 2). In general, the genomic similarity of strain G7^T^ with *Methylobacter* spp. isolates and MAGs assembled from enrichment cultures varied between 77.2–95.7% ANI, 70.1–94.7% AAI and 20.5–63.9% dDDH (Table 1). Considering the widely applied thresholds for 16S rRNA gene identity (<98.65%) [31,32], ANI (<95%) and dDDH (<70%) [19,32,35], as well as a previously suggested threshold for *pmoA* gene identity (<87 % and <93 % at nucleotide and amino acid levels, respectively) [36] to delineate unique species, strain G7^T^ represents a novel species. Based on MAG data, *Ca*. M. titanis would represent a closely related but different species, since dDDH between it and strain G7^T^ was also lower than 70%. Despite sharing 16S rRNA and *pmoA* gene identities higher than the species-level threshold with some isolates of *Methylobacter* spp. (Table 1), the genome-level differences, i.e. ANI and dDDH, of strain G7^T^ with other isolates were clearly lower than the species-level thresholds (Table 1). Altogether, this data suggests that strain G7^T^ represents a novel species of *Methylobacter* spp., for which G7^T^ is the only currently existing isolate. It has to be noted, however, that the 16S rRNA and *pmoA* gene-based phylogenetic trees and the phylogenomic tree reveal that the genus *Methylobacter* as currently circumscribed is polyphyletic (Fig. 1), as was also previously reported [34,37]. Hence, the clade including *M. svalbardensis* LS7-T4A^T^, *Ca*. M. titanis and strain G7ᵀ may warrant recognition as a coherent subclade, or even a separate genus, pending possible broader taxonomic revision in the future, which, however, is out of the scope of this paper.

**Fig. 2.**
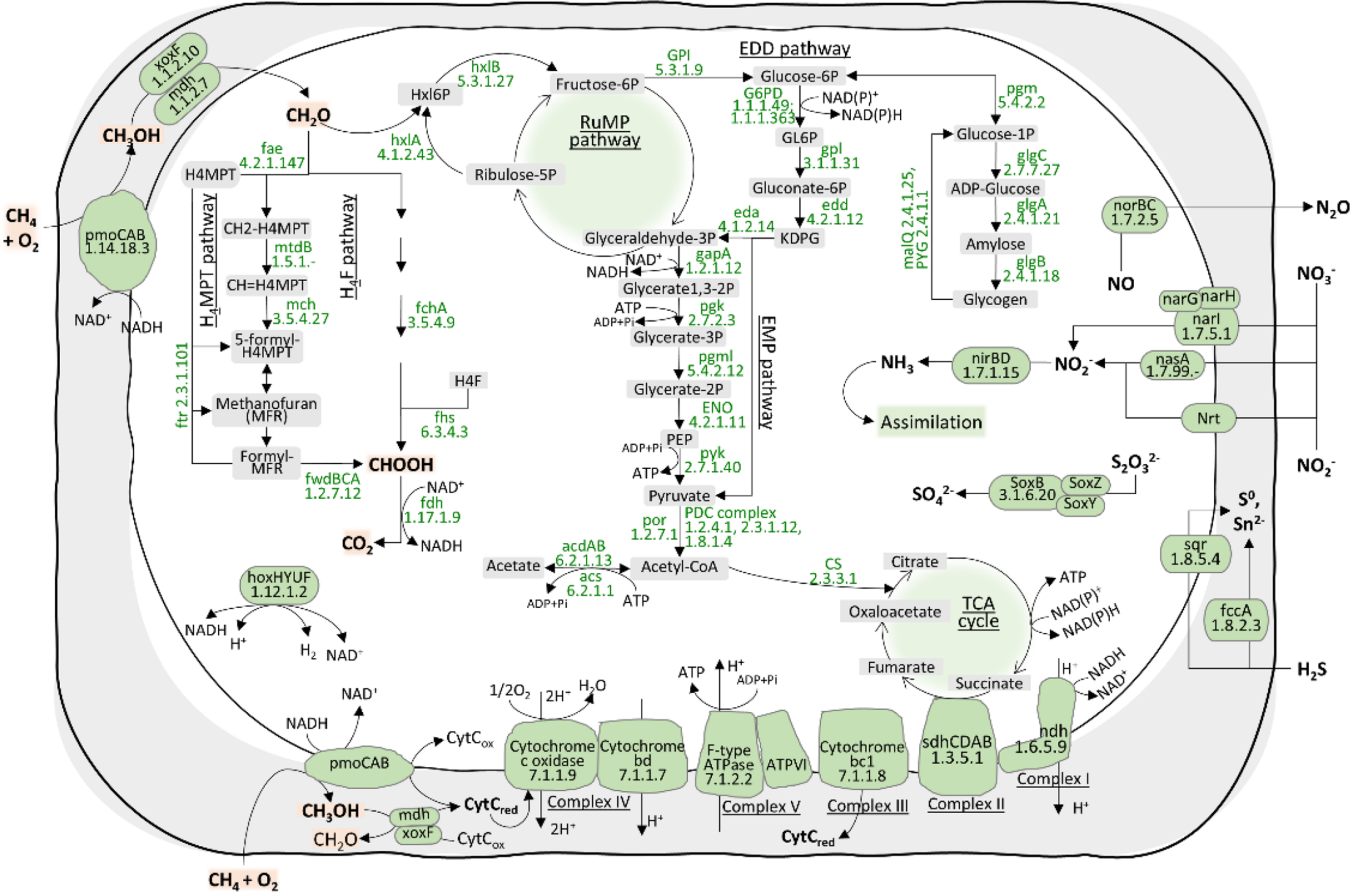
The proposed central metabolic pathway demonstrated carbon assimilation and energy metabolisms of *Methylobacter arcticus* G7^T^ based on the assembled genome. H_4_MPT, tetrahydromethanopterin; H_4_F, methylene tetrahydrofolate; KDPG, 2-keto-3-deoxy-6-phosphogluconate; GL6P, d-glucono-1,5-lactone 6-phosphate; Hxl6P, d-arabino-3-hexulose 6-phosphate; PEP, phosphoenolpyruvate; PDC, pyruvate dehydrogenase complex; EDD, Enter–Doudoroff; EMP, Embden–Meyerhof–Parnas. The list of functional gene details is provided in Table S1.

The key metabolic pathways present in the genome of strain G7^T^ were predicted using METABOLIC (v. 4.0) based on the KEGG database [13] (Fig. 2, Table S1). Strain G7^T^ contains genes encoding particulate methane monooxygenase (pmoCAB) but not soluble methane monooxygenase (mmoXYZBCD) for methane oxidation, unlike *Methylobacter* sp. S3L5C and *M. psychrophilus* Z-0021^T^ (Fig. 2, Tables 2 and S1). Additionally, several other *Methylobacter* spp. contain the pxmABC operon, i.e. a copper membrane monooxygenase [22], but it is absent in strain G7^T^ (Table 2). For the conversion of methanol to formaldehyde, strain G7^T^ contains both calcium- (mxaFJGIACKLD) and lanthanide-dependent (xoxF) methanol dehydrogenases (Fig. 2, Tables 2 and S1). All essential genes involved in both tetrahydromethanopterin (H_4_MPT) and tetrahydrofolate (H_4_F) pathways are present for formaldehyde oxidation to formate. For formate oxidation to CO_2_, formate dehydrogenases (fdh, fdhF and fdsD) are present in the genome. Strain G7^T^ possesses a complete ribulose monophosphate cycle for formaldehyde assimilation into biomass, while the serine pathway is incomplete. Furthermore, it contains all functional genes encoding functions of Entner–Doudoroff, Embden–Meyerhof–Parnas, oxidative TCA pathways and oxidative phosphorylation for sugar metabolism and energy production. Several genes involving an incomplete denitrification pathway are present in the strain G7^T^’s genome, including nitrate reductase (narGHI) for nitrate reduction into nitrite, and nitric oxide reductase (norBC) for nitric oxide reduction to N_2_O, while nitrite reductases (nirKS) and nitrous-oxide reductase (nosZ) are absent. Strain G7^T^ contains genetic potential for oxidation of sulphur compounds, such as hydrogen sulphide (H_2_S) into elemental sulphur and polysulphides using sulphide-quinone oxidoreductase (sqr) and cytochrome subunit of sulphide dehydrogenase (fccA). It also carries the potential to oxidize thiosulphate to sulphate using the Sox enzyme complex, i.e. SoxB and SoxYZ. The analysis of secondary metabolite biosynthesis genes revealed genetic potential to produce aryl polyene, ectoine, non-ribosomal peptide synthetase (NRPS), NRPS-like fragments, other unspecified ribosomally synthesized and post-translationally modified peptide product, NRPS-independent IucA/IucC-like siderophores (NI-siderophores), terpene precursors, terpene and redox cofactors.

**Fig. 3.**
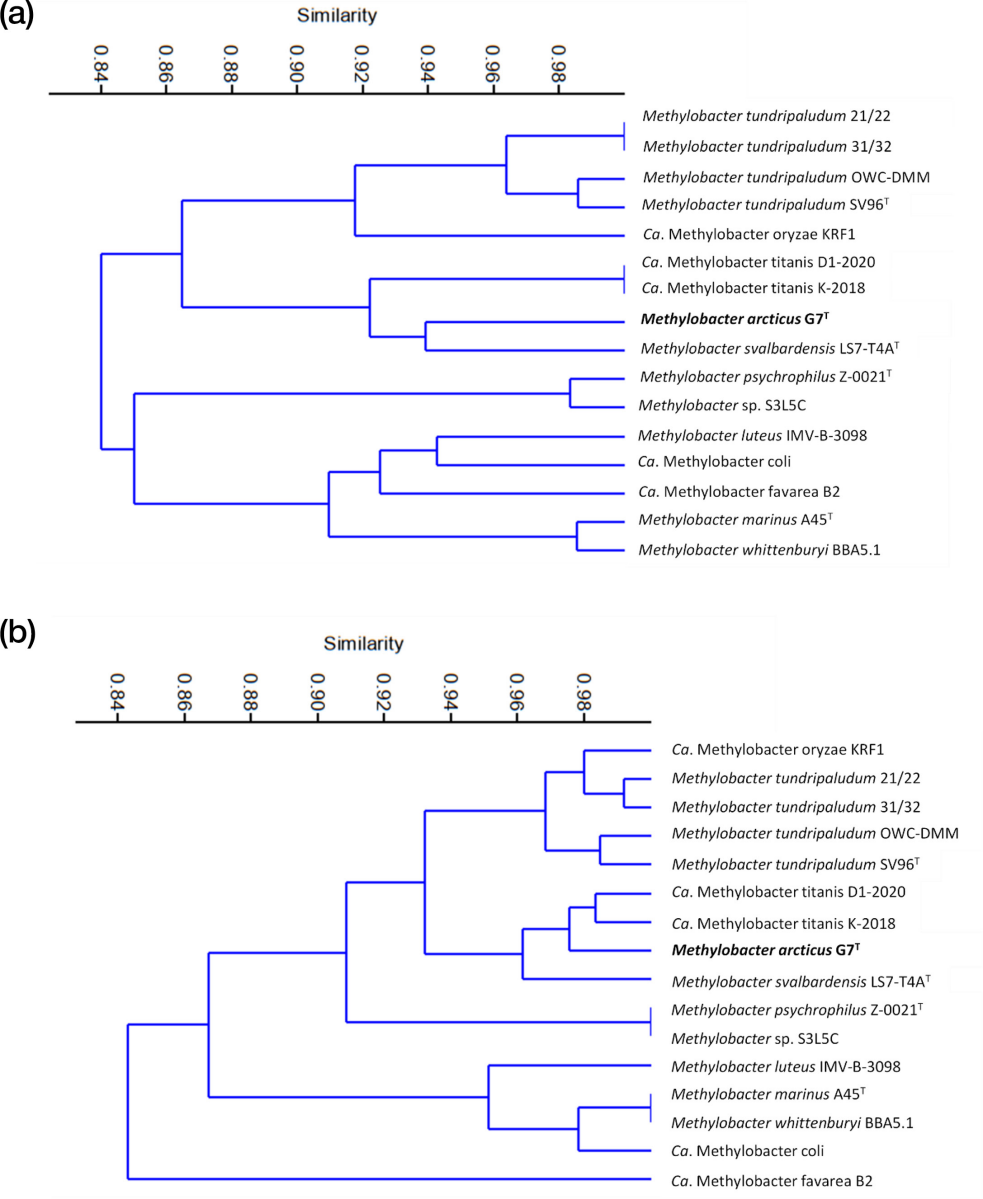
Similarity of functional potential among *Methylobacter* spp. as visualized through UPGMA clustering (Bray–Curtis dissimilarity index) based on presence/absence table of (a) functional traits and (b) KEGG modules.

The general differences in the functional potential between *Methylobacter* spp. were assessed via UPGMA cluster analysis of the presence–absence data of functional traits and KEGG modules encoded in the genomes (data generated using METABOLIC [13]) (Fig. 3). Similar to phylogenetic and phylogenomic tree analyses, strain G7^T^ was most similar to *Ca*. M. titanis and *M. svalbardensis*, suggesting that they also functionally differed from other *Methylobacter* spp. (Fig. 3). The clustering of *Methylobacter* spp. based on the functional potential correlated with the phylogenetic and phylogenomic trees (compare Figs1  3).

To further show that strain G7^T^ is a novel species of *Methylobacter*, its functional potential was compared with all other *Methylobacter* spp. Exceptionally, in contrast to all other *Methylobacter* spp. isolates, strain G7^T^ did not encode nitrogenase enzyme (nifDKH), essential for N_2_ fixation (Table 2). MAGs representing *Ca*. M. titanis also lacked N_2_ fixation genes. The analysis of secondary metabolites revealed that besides G7^T^, the genes encoding ectoine production were found only in MAG representing *Ca*. M. titanis D1-2020, and in genomes of *M. marinus* A45^T^ and *Methylobacter* sp. BBA5.1 (here representing the genome of *M. whittenburyi* since the genome of the type strain was not available).

The functional potential of strain G7^T^ was specifically compared to the closest isolate, *M. svalbardensis* LS7-T4A^T^, using KEGG annotations from KofamKOALA. The number of shared genes with KO classification was 1,524 between G7^T^ and LS7-T4A^T^, while G7^T^ had 98 and LS7-T4A^T^ had 205 unique KO-classified genes in their genomes (Fig. S2). Besides the lack of N_2_ fixation potential, compared to LS7-T4A^T^, G7^T^ lacked genetic potential to e.g. NADH-quinone oxidoreductase (nuoA-N), cyanate lyase (cynS) and hydroxylamine reductase (hcp). Furthermore, strain G7^T^ encoded a larger number of potential biofilm formation and carotenoid biosynthesis genes (i.e. 20 and 5 genes, respectively) than LS7-T4A^T^ (i.e. 14 and 1 genes, respectively), while LS7-T4A^T^ encoded a larger number of potential quorum-sensing genes (11 genes) than G7^T^ (5 genes). In addition, METABOLIC results suggested that strain G7^T^ and LS7-T4A^T^ differed in their genetic potential for iron cycling. Both had the *cyc1* gene encoding iron oxidation and *FmnB*, *Ndh2* and *DmkB* genes encoding dissimilatory iron reduction, but in contrast to LS7-T4A^T^, strain G7^T^ lacked the *MtoA* gene encoding iron oxidation and several other genes encoding dissimilatory iron reduction (*MtrA*, *MtrB_TIGR03509*, *DFE_0448*, *DFE_0449*, *DFE_0461* and *DFE_0462*; see Garber *et al*. [38] and references therein for further information on these genes). The genetic potential for secondary metabolite production was otherwise similar between G7^T^ and LS7-T4A^T^ (see above the listing for G7^T^), except for the lack of ectoine production genes in LS7-T4A^T^. The exceptional lack of N_2_ fixation potential in strain G7^T^ compared to other *Methylobacter* spp., and the other differences in functional potential revealed in the comparison of LS7-T4A^T^ and strain G7^T^, further supports the assignation of strain G7^T^ as representative of a novel *Methylobacter* species.

To explain if the observed genetic differences could be linked to habitat adaptation of G7^T^ and LS7-T4A^T^, further physicochemical characterizations of the isolation habitats and metagenomic screening and experimental studies would be needed. However, some testable hypotheses on their potential ecological differences can be made based on genetic data. The larger number of biofilm formation genes in the genome of G7^T^ likely predicts its lifestyle as biofilm-forming cells on the coal surface, whereas LS7-T4A^T^ more likely lives as free cells in the aquatic sediment. Furthermore, as carotenoids act as antioxidants [39], and ectoine is a natural osmo-protectant produced by many bacterial species to survive extreme environmental conditions like high salinity, temperature and drought [40], it can be predicted that the larger number of these genes in the genome of G7^T^ reflects its higher need for stress tolerance mechanism in the coal mine. The higher number of genes encoding dissimilatory iron oxidation and reduction in the genome of LS7-T4A^T^ may reflect habitat differences, i.e. higher availability of oxidized and reduced iron in the aquatic sediment with a natural redox gradient than on the coal surface.

### Morphological, physiological and chemotaxonomic characteristics

**Fig. 4.**
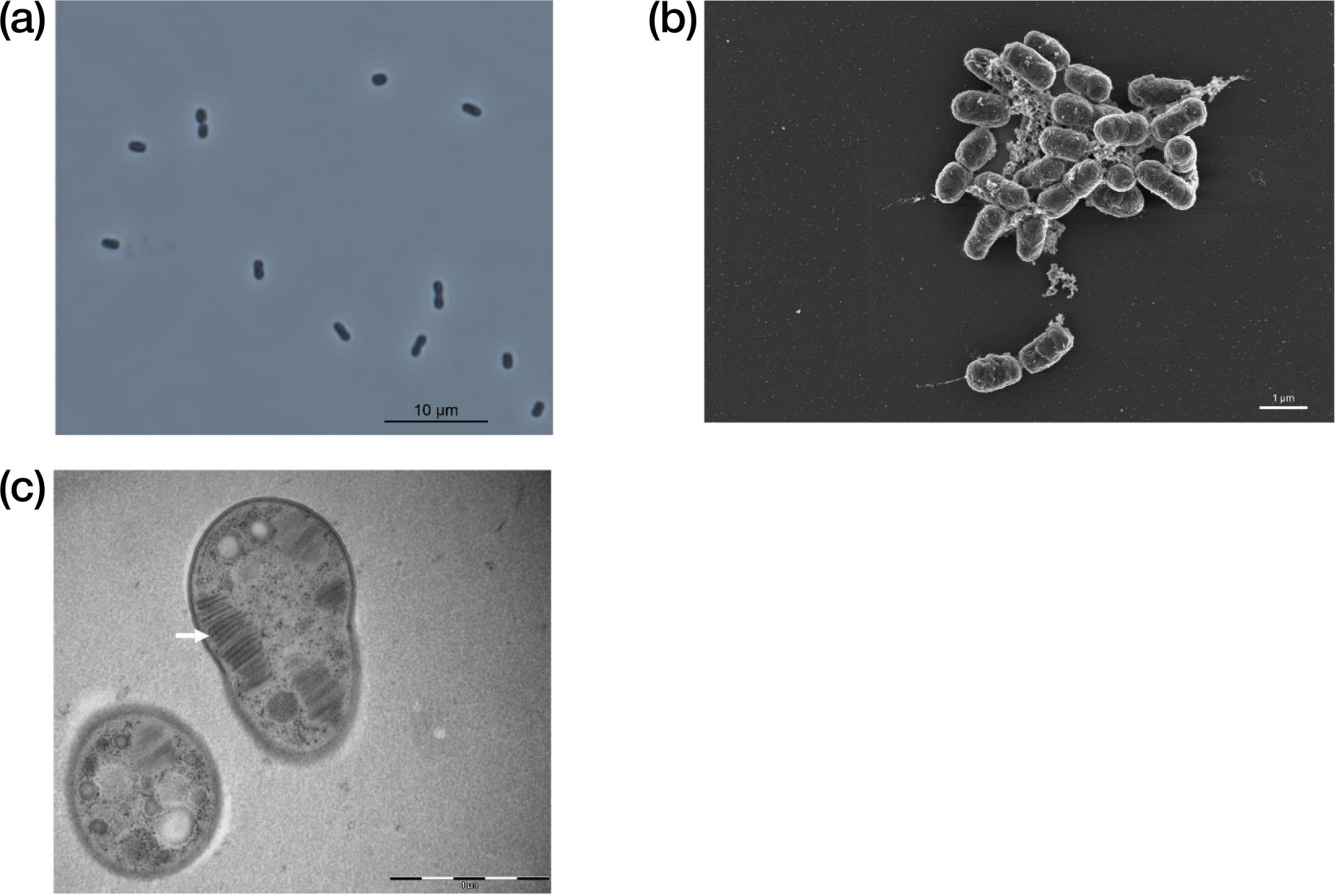
Morphological characteristics of strain G7^T^. (**a**) Phase-contrast microscopy image of living cells. (**b**) SEM image of cells. (**c**) TEM image of ultrathin sections of the cells. The white arrow indicates the intracytoplasmic membranes (ICMs) arranged in stacks.

The cells of strain G7^T^ were Gram-stain-negative, motile rods (0.8–1.0×1.5–2.0 µm) (Fig. 4). Cells reproduced by binary fission. The strain was shown to grow at 4–21 °C with optimum growth at 8 °C (Table 3) [8]. It did not grow at +23 °C or +27 °C. The growth was not tested at temperatures lower than +4 °C [8]. Strain G7^T^ did not grow in 0% methanol, but there was growth at methanol concentrations from 0.01 to 1%, with increasing growth at higher concentrations. Nitrate and ammonia supported growth equally well, whereas no growth was detected when N_2_ was the only available nitrogen source. NaCl at 0.5% was tolerated, 1% inhibited growth, and above 2% NaCl, no growth was detected. The optimal pH of G7^T^ was 6–7, with a growth range of approximately pH 5 to 9.

**Table 3.** Comparison of major characteristics of strain G7^T^ with its phylogenetically closest relatives

	*M. arcticus* G7^T^	*M. svalbardensis* LS7-T4A^T^	*Ca*. M. titanis	*Methylobacter* sp. S3L5C	*M. psychrophilus* Z-0021^T^	*M. tundripaludum* SV96^T^
**Cell morphology**	Rod	Coccoid to rod	Cocci	Cocci	Cocci	Rods
**Cell size (µm)**	0.8–1.0×1.5–2.0	0.8–1.2×1.6–2.2	nd	1.0–1.8 in diameter	1.0–1.7 in diameter	0.8–1.3×1.9–2.5 With characteristic four-cell chains
**Motility**	Yes	Yes, some	nd	No	No	Yes, some
**Pigmentation**	Pink	Light pink	nd	Cream	Pinkish	Pale pink
**Growth T (optimal)**	4–21 °C (8 °C)*	1–22 °C (10–15 °C)	5–30 °C (15 °C)†	0.1–20 °C (8–12 °C)	1–20 °C (3.5–10 °C)	5–30 °C (23 °C)
**Growth pH (optimal)**	5–9 (6–7)	6.4–9.3 (7.5–8)	nd	6–8.3 (6–7.3)	5.9–7 (6.7)	5.5–7.9
**Growth on nitrate (N) vs ammonium (A)**	Both, equally good	N	nd	Both, A best	Both, A best	Both, N best
**Growth on N_2_**	nd (no nitrogenase gene detected)	nd	nd	No	nd	nd
**Growth on methanol**	Yes	Yes	nd	Yes	Yes	No, poorly
**Major PFLA(s)**	16 : 1ɷ7c, 16 : 1ɷ5c	nd	nd	nd	16 : 1	16 : 1ɷ8, 16 : 1ɷ7, 16 : 1ɷ5t
**NaCl tolerance (inhibition level in NMS)**	>1% w/v	>0.5% w/v	nd	nd	nd	>0% w/v
**Source**	Arctic coal mine biofilm	Arctic Lagoon Pingo sediments	Antarctic lake sediment	Boreal lake water	Arctic tundra soil	Arctic wetland soil
**Reference**	This study and Tveit *et al*. [8]	Patil *et al*. [22]	Roldán and Menes [25]	Khanongnuch *et al*. [26]	Omelchenko *et al*. [23,24]	Wartiainen *et al*. [27]

*Tested in six temperatures: +4 °C, 8 °C, 15 °C, 21 °C, 23°C and 27 °C. G7 does not grow at 23 °C or 27 °C.

†Data from enrichment culture. *Ca*. M. titanis has not been isolated.

Strain G7^T^ had a lower optimum growth temperature than its close relatives, *M. svalbardensis* LS7-T4A^T^, *Ca*. M. titanis and *M. tundripaludum* SV96^T^, and *Ca*. M. titanis and SV96^T^ grew at higher temperatures than G7^T^ (Table 3). The growth temperature range and optimum temperature of G7^T^ were similar to those of *Methylobacter* sp. S3L5C and *M. psychrophilus* Z-0021^T^, yet cell morphologies differed (Table 3). G7^T^ could grow at a lower pH than its close relatives, and its optimum growth pH was also lower than that of LS7-T4A^T^ (Table 3). The PLFA profile of G7^T^ also differed from that of SV96^T^ [8]. Comparison of PLFA profiles of G7^T^ to those of LS7-T4A^T^, S3L5C and Z-0021^T^ cannot be done due to the lack of data for LS7-T4A^T^ and S3L5C, and for Z-0021^T^, it is only reported that its main PLFA is 16 : 1 [23].

In conclusion, based on phenotypic, genetic and phylogenetic data, strain G7^T^ represents a novel species within the genus *Methylobacter* for which the name *Methylobacter arcticus* sp. nov. is proposed.

## Description of *Methylobacter arcticus* sp. nov.

*Methylobacter arcticus* (arc’ti.cus. L. masc. adj. *arcticus*, northern, pertaining to the Arctic). Cells are Gram-stain-negative, motile rods with a diameter of ~0.8–1.0 µm and 1.5–2.0 µm long. The cell possesses a gammaproteobacterial ICM structure. They form circular pink colonies with even edges and do not produce water-soluble pigments. Cultivation is done in NMS medium (DSMZ medium 921, pH 6.8) with a headspace consisting of 20% methane (v/v) in air. Carbon and energy sources are methane and methanol. The strain can use both nitrate and ammonium but does not fix nitrogen and does not require vitamins. It tolerates a broad temperature range from <4 °C to 21 °C, with optimal growth at 8 °C and pH 6–7.

PLFAs are C12 : 0, C14 : 0, C15 : 0, C16 : 1 ω11c, C16 : 1 ω7c, C16 : 1 ω5c, C16 : 0 and C18 : 1 ω7c, of which the major ones (>10%) are C16 : 1 ω7c (58.1%) and C16 : 1 ω5c (16.7%). The G+C content is 47.7 mol%. The GenBank accession numbers for the genome and the 16S rRNA gene sequence are CP159583 and PV005874, respectively. The raw sequence reads of the genome are stored in Short Read Archive under BioProject PRJNA1127878.

The type strain is G7^T^ (=DSM 117899^T^= LMG 33632^T^) which was isolated from the coal mine Gruve 7, in Adventdalen, Svalbard, Norway.

## Supplementary material

10.1099/ijsem.0.006984 Uncited Supplementary Material 1.

10.1099/ijsem.0.006984 Uncited Supplementary Material 2.

## Abbreviations

AAI: average amino acid identity
ANI: average nucleotide identity
dDDH: digital DNA–DNA hybridization
gDNA: genomic DNA
ICMs: intracytoplasmic membranes
MAG: metagenome-assembled genome
NMS: nitrate mineral salts
NRPS: non-ribosomal peptide synthetase
PLFAs: phospholipid fatty acids
SEM: scanning electron microscopy
TEM: transmission electron microscopy
TYGS: Type Strain Genome Server
