# Supplementary material for: Methylobacter arcticus sp. nov. isolated from a coal mine biofilm in the high Arctic Svalbard
Source: Int J Syst Evol Microbiol. 2025 Nov 28;75(11):006984. doi: 10.1099/ijsem.0.006984 (PMC12662621; doi:10.1099/ijsem.0.006984)
Supplement: Uncited Supplementary Material 1. [file ijsem-75-06984-s001.pdf]

Supplementary material of this manuscript includes:

- **Supplementary figures Figure S1 and Figure S2 (included in this file)**
- **Supplementary Table S1 (included in a separate .xlsx – file)**

Figure S1

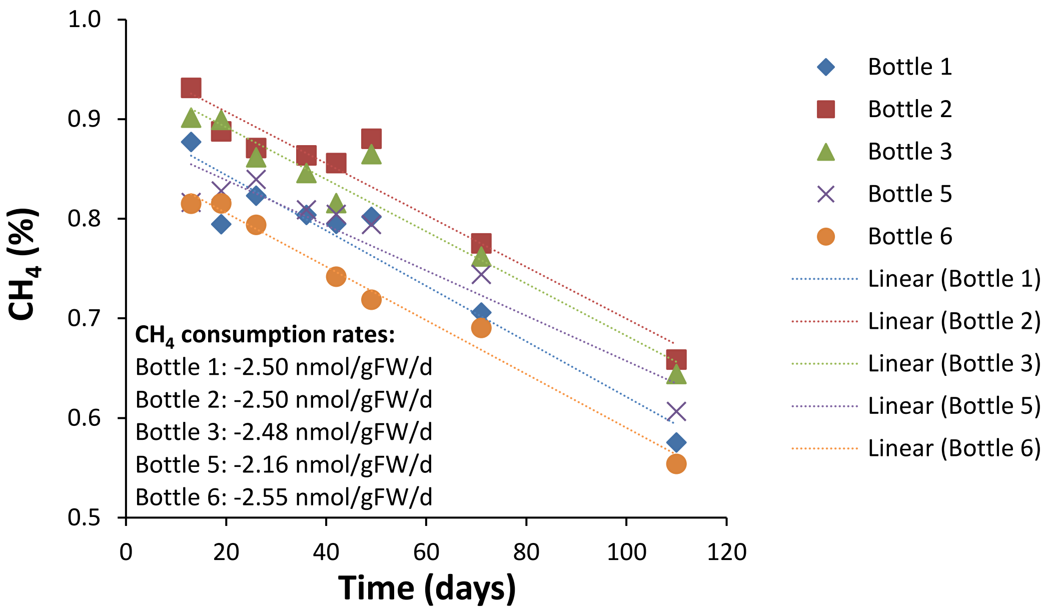

Figure S1. Methane consumption of coal between days 13 and 110 of incubation. Broken coal (total weight 387.2g) was collected from Gruve 7 mine, transported at 4-10°C to the lab, and broken further to pieces (≤ 1 cm). Coal (ca. 50 g) was incubated in serum bottles at 10°C in the dark and amended with methane. Headspace methane decreased linearly with time between 13 and 110 days of incubation. One bottle (Bottle 4) out of six was lost. Methane consumption rates were calculated between days 13 and 110, when decrease of methane was constant with time.

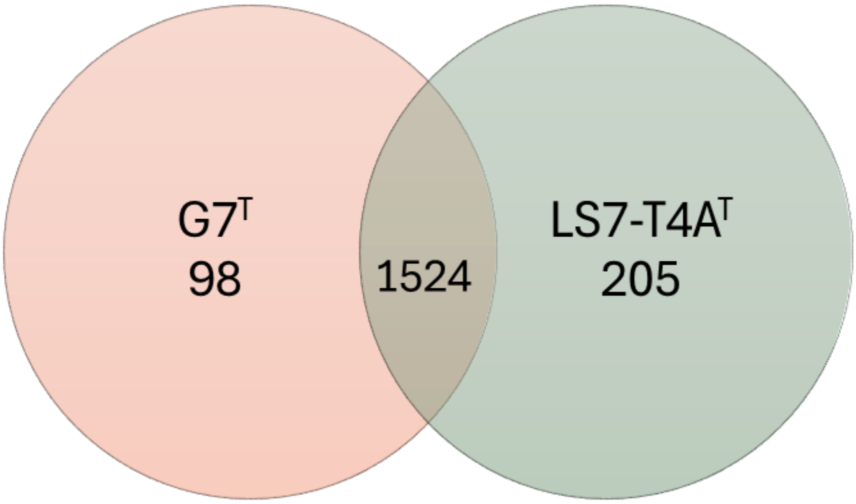

Figure S2. Venn diagram showing the number of shared and unique genes having KEGG Orthology (KO) classification in the genomes of *Methylobacter arcticus* G7^{T} and *Methylobacter svalbardensis* LS7-T4A^{T}.
